# Engineered Human Ferritin Nanoparticles for Direct Delivery of Tumor Antigens to Lymph Node and Cancer Immunotherapy

**DOI:** 10.1038/srep35182

**Published:** 2016-10-11

**Authors:** Bo-Ram Lee, Ho Kyung Ko, Ju Hee Ryu, Keum Young Ahn, Young-Ho Lee, Se Jin Oh, Jin Hee Na, Tae Woo Kim, Youngro Byun, Ick Chan Kwon, Kwangmeyung Kim, Jeewon Lee

**Affiliations:** 1 Department of Chemical and Biological Engineering, Korea University, Anam-Dong 5-1, Seongbuk-Gu, Seoul 136-713, Republic of Korea; 2 Center for Theragnosis, Biomedical Research Institute, Korea Institute of Science and Technology, 39-1 Hawolgok-dong, Seongbuk-gu, Seoul 136-791, Republic of Korea; 3 Department of Molecular Medicine and Biopharmaceutical Sciences, Graduate School of Convergence Science and Technology, Seoul National University, Seoul 151-742, Republic of Korea; 4 Division of Infection and Immunology, Graduate School of Medicine, Korea University, Anam-dong 5-1, Seongbuk-gu, Seoul 136-701, Republic of Korea

## Abstract

Efficient delivery of tumor-specific antigens (TSAs) to lymph nodes (LNs) is essential to eliciting robust immune response for cancer immunotherapy but still remains unsolved. Herein, we evaluated the direct LN-targeting performance of four different protein nanoparticles with different size, shape, and origin [*Escherichia coli* DNA binding protein (DPS), *Thermoplasma acidophilum* proteasome (PTS), hepatitis B virus capsid (HBVC), and human ferritin heavy chain (hFTN)] in live mice, using an optical fluorescence imaging system. Based on the imaging results, hFTN that shows rapid LN targeting and prolonged retention in LNs was chosen as a carrier of the model TSA [red fluorescence protein (RFP)], and the flexible surface architecture of hFTN was engineered to densely present RFPs on the hFTN surface through genetic modification of subunit protein of hFTN. The RFP-modified hFTN rapidly targeted LNs, sufficiently exposed RFPs to LN immune cells during prolonged period of retention in LNs, induced strong RFP-specific cytotoxic CD8^+^ T cell response, and notably inhibited RFP-expressing melanoma tumor growth in live mice. This suggests that the strategy using protein nanoparticles as both TSA-carrying scaffold and anti-cancer vaccine holds promise for clinically effective immunotherapy of cancer.

Immunotherapy is an emerging and promising new approach to treatment of cancer. Cancer vaccine, a type of cancer immunotherapeutic agent aims to activate or enhance body’s adaptive immune system to effectively recognize and kill tumor cells. Tumors express a variety of specific antigens, and the tumor-associated antigens comprise an indispensable part of cancer vaccine that induces robust and tumor-specific immune response to fight the cancer^1^,^2^,^3^. Upon encountering tumor-associated antigens, dendritic cells (DCs) are activated into antigen-presenting cells (APCs) that allow for the cross-presentation of antigens to T cells. This induces the production of tumor antigen-specific cytotoxic CD8^+^ T cells, which have the ability to kill tumor cells^4^,^5^,^6^. In this regard, LNs can be a strategic target for antigen delivery, because LNs contain a high population of resident DCs, plasmacytoid DCs, and macrophages^7^,^8^ that can be converted to APCs. Free tumor antigens, including proteins and peptides from tumor cells, are quickly cleared before they reach LNs^9^ and mostly ignored by the immune system^10^. Therefore, the development of antigen carrier systems capable of targeting LNs and eliciting a strong immune response is vital for efficient cancer immunotherapy.

According to the previous results^7^,^10^,^11^,^12^,^13^,^14^,^15^,^16^,^17^,^18^, antigen delivery through lymphatic system to LNs was enhanced when antigens were carried by synthetic nanoparticles (NPs), and the immunogenic activity of NP-associated antigens was improved compared to free individual antigens because NPs can deliver many antigens owing to their enhanced surface to volume ratio^19^ and are easily taken up by DCs. The antigen delivery to LNs by synthetic NPs is highly dependent on NP size: large NPs (>100 nm) remain almost confined to the injection area, whereas small molecules (<20 kDa) or small NPs (<10 nm) rapidly and freely enter systemic circulation via bloodstream, and the NPs in size of 10–80 nm can migrate to LNs through lymphatic system and are considered optimal for LN targeting. However, despite the appreciable advantages of synthetic NPs as cancer nanomedicines, only a few such NPs have shown promising therapeutic outcomes and advanced to clinical trial stages^20^,^21^,^22^. One of main factors likely to hamper the clinical feasibility of synthetic NPs is intrinsic cytotoxicity and nanotoxicity associated with *in vivo* accumulation of non-degradable synthetic NPs^23^,^24^,^25^,^26^,^27^. Accordingly only a few biomaterial-based particles (e.g. albumin-derived Abraxane^®^) have been clinically approved^20^.

It is worth noting that protein-based nano-scale particles (named “protein nanoparticles” here) that are self-assembled inside cells to form 3D nanostructures and afford well-oriented architecture with constant shape, size, and surface topology^28^,^29^,^30^ can be engineered to expose specific proteins or peptides on their surface through genetic modification of internal region or N- or C-terminus of protein subunit^24^,^26^,^28^,^29^,^30^,^31^,^32^,^33^. This suggests that protein nanoparticles can be excellent molecular scaffolds to carry tumor-associated antigens. Also, surface-exposed amines, thiols, and carboxylates on protein nanoparticles are effective for conjugating various imaging agents including fluorescent dyes and isotopes. In addition, protein nanoparticles are spontaneously disassembled in living organisms and then degraded or removed through renal excretion within several days, showing excellent biocompatibility^24^.

As illustrated in Fig. 1, we evaluated the efficacy of four candidate protein nanoparticles with different origin, size, and shape in targeting LNs: 1) *Escherichia coli* DNA binding protein (DPS), 2) *Thermoplasma acidophilum* proteasome (PTS), 3) hepatitis B virus capsid (HBVC), and 4) human ferritin heavy chain particle (hFTN), found that hFTN was preferentially delivered to and sufficiently accumulated in LNs, and selected hFTN as a carrier of the model tumor antigen, RFP. After efficiently delivered to LNs, the genetically modified hFTN (hFTN-RFP) that densely presents RFP on the hFTN surface induced RFP-specific immunogenic activity against RFP-expressing B16F10 melanoma tumor cells and successfully inhibited the tumor growth in live mice.

## Results

### Biosynthesis of candidate protein nanoparticles with different origin, size, and shape

**Figure 2:**
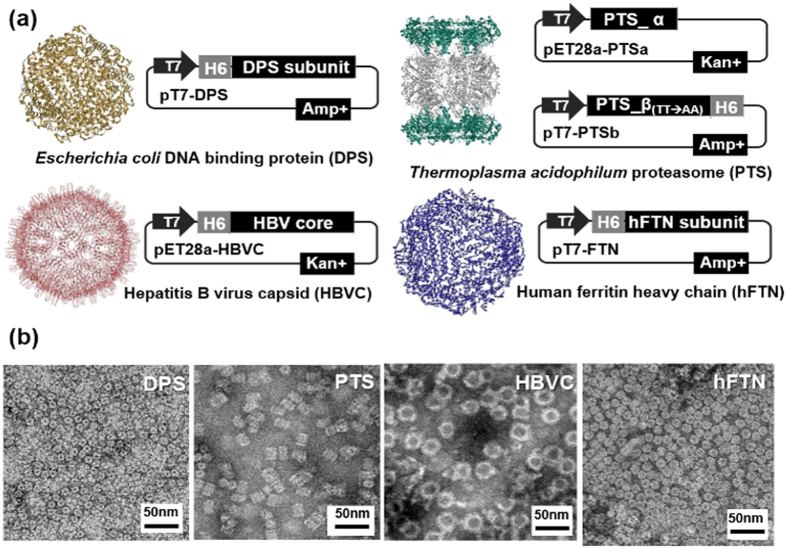
Biosynthesis of protein nanoparticles. (**a**) 3D shape of 4 protein nanoparticles (DPS, PTS, HBVC, and hFTN) tested in LN targeting and plasmid expression vectors used for the biosynthesis of protein nanoparticles in *E. coli*. (In case of PTS, *E. coli* was transformed with two expression vectors to simultaneously express two different protein subunits, PTS-α and PTS-β). (**b**) TEM images of purified protein nanoparticles.

When expressed using plasmid expression vectors in *E. coli*, the protein subunits of DPS, PTS, HBVC and hFTN were synthesized and subsequently self-assembled to form 4 different protein particles with different shape, size, and surface property in the cytoplasm of *E. coli* (Fig. 2, Table 1). Sodium dodecyl sulfate-polyacrylamide gel electrophoresis (SDS-PAGE) of the purified DPS, HBVC, and hFTN revealed a single protein band corresponding to the respective protein subunit, and the two protein bands for PTS correspond to the two different subunits, PTS-α and PTS-β (Supplementary Fig. 2). All the protein nanoparticles have native-like particle shape and size with narrow size distribution, as confirmed by transmission electron microscopy (TEM) (Fig. 2b) and dynamic light scattering (DLS) analyses (Table 1). The diameters of spherical DPS, hFTN, and HBVC were 9.5 ± 1.2, 11.7 ± 0.8, and 32.3 ± 1.9 nm, respectively. The cylindrical PTS (native size: 11 × 15 nm with a stacked four-ring structure) was 13.4 ± 2.1 nm. The size of all the protein nanoparticles (ranging from 9.5 to 32.3 nm) fulfills the prerequisites for *in vivo* LN targeting (10 to 80 nm)^10^,^11^,^13^,^14^,^15^. Moreover, all the protein nanoparticles presented a slightly negative Zeta potential charge (Table 1), indicating that the surface charge effect on targeting immune cells in LN is all equal for the four different protein nanoparticles here.

### Selection of optimum protein nanoparticle in directly targeting LNs of mice

**Figure 3:**
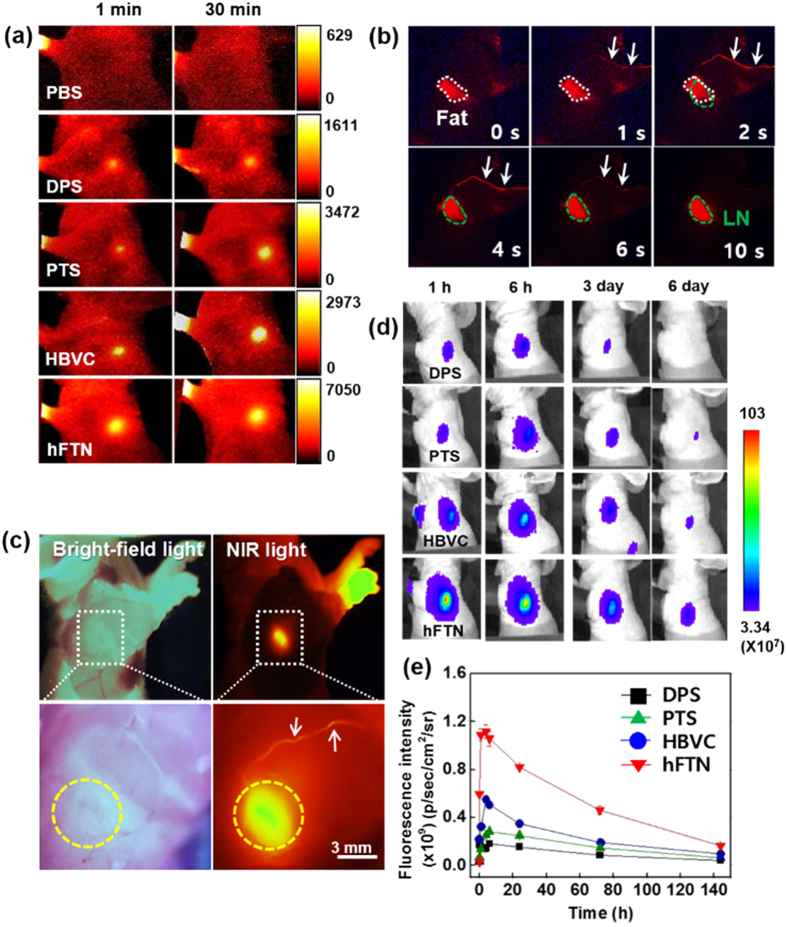
Direct LN targeting of protein nanoparticles that are Cy5.5-labeled and subcutaneously injected through the footpad of C57BL/6 mice. (**a**) *In vivo* near-infrared (NIR) fluorescence images acquired with a Kodak image station at pre-determined time points. (**b**) Real-time intravascular dynamics of hFTN, observed in a skin flap of live mice. [The white arrows indicate rapid migration of hFTN to LNs (inside green dotted lines) through lymphatic vessels. LN signal differentiates it from the auto-fluorescence of fatty tissues (inside white dotted lilnes) around the LN.] (**c**) LN images of mice (**b**) with removed skin under bright-field light and under NIR light excitation. (**d**,**e**) Long-term NIR fluorescence images and intensity, 2-times measured by IVIS spectrum imaging system at pre-determined time points for 6 days.

In order to estimate the *in vivo* LN-targeting efficiency of four different protein nanoparticles in live animal, protein nanoparticles were labeled by a NIRF dye (Cy5.5, 0.1 wt%) in PBS and properly diluted to make all the protein nanoparticle solutions have the same NIRF intensity (Supplementary Fig. 2), followed by injection (100 μl) into the left footpad of healthy mice. No signal was detected in the LNs in the case of PBS injection (Fig. 3a). Within 1 min post injection, NIRF signals of LNs were clearly detected and increased for 30 min in the injection of protein nanoparticles, and in particular, the LN NIRF signal in hFTN-treated mice was obviously the highest among the four protein nanoparticles (Fig. 3a, Supplementary Fig. 3). Also the real-time lymphatic vessel dynamics indicates the fast migration of hFTN to the target LNs (Fig. 3b,c). The long-term analysis of NIRF images of LNs shows that the hFTN produced always 2 to 6-fold stronger LN fluorescence signal up to 6 days after injection than the other protein nanoparticles and a clearly visible LN signal even at 6^th^ day, indicating the significantly prolonged retention of hFTN in LNs (Fig. 3d,e). From the real-time lymphatic vessel dynamics of hFTN, observed in skin flaps of live mice using the Olympus OV100 imaging system (Fig. 3b), hFTN rapidly migrated to the LNs through lymphatic vessels (indicated by white arrows) within just 1–4 sec post injection and generated strong NIRF signal in the LNs (green dotted circles) within 10 sec, differentiating it from the auto-fluorescence of fatty tissues (white dotted circles) that are located near LNs. Also from Fig. 3c, the LN NIRF signal (yellow dotted circles) was obviously identified under NIR light excitation after 1 h post injection of hFTN, whereas the LNs were not visible under bright-field light, indicating the LN-specific targeting of hFTN. Enhanced accumulation of hFTN in LNs was also confirmed by *ex vivo* fluorescence imaging (Supplementary Fig. 4a), showing that the NIRF signals were detected only in the proximal LNs (injection side), but not in the distal LNs, indicating locally targeted delivery of the protein nanoparticles after subcutaneous injection. The hFTN-treated LNs exhibited the highest NIRF signal, i.e. 3 to 7-fold higher than the other protein nanoparticle-treated LNs (Supplementary Fig. 4b). Moreover, from the *ex vivo* NIRF images of excised organs (liver, lung, spleen, kidney, heart, and LNs), the highest NIRF signals were clearly observed in the hFTN-treated LNs (Supplementary Fig. 4c). Histological fluorescence imaging of the dissected LNs at 24^th^ h post injection also indicates the brightest fluorescence signals of hFTN-treated LNs (Supplementary Fig. 4d). Based on the results of Fig. 3 and Supplementary Figs 3 and 4, hFTN was selected as a preferable carrier of tumor antigen to elicit robust response of immune cells in LNs.

### Engineering of human ferritin nanoparticle (hFTN) to present model TSA (RFP) on hFTN surface

**Figure 4:**
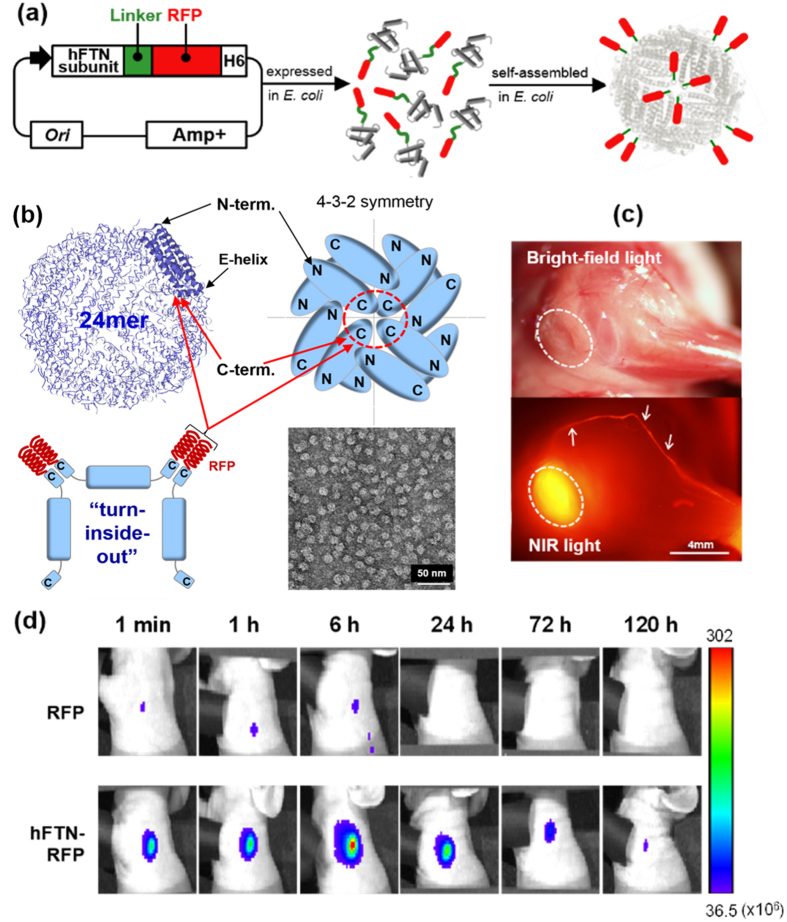
Model tumor antigen (RFP)-displaying hFTN (hFTN-RFP) as a cancer immunotherapeutic vaccine. (**a**) Schematic illustration of biosynthesis of hFTN-RFP in *E. coli*, affording surface display of RFPs on hFTN. (**b**) Characteristic surface structure of hFTN (PDB ID: 3AJO) and surface engineering of hFTN to display RFPs. (TEM image shows purified hFTN-RFP. N, E, and bold blue part indicates N-terminus, C-terminal E-helix, and entire subunit protein of hFTN, respectively.). (**c**) LN images of mice with removed skin under bright-field light and under NIR light excitation after subcutaneous injection of hFTN-RFP to mice. (**d**) Long-term NIR fluorescence images of C57BL/6 mice at pre-determined time points after subcutaneous injection of RFP and hFTN-RFP through the footpad of mice.

To develop a ferritin nanoparticle-based cancer vaccine, RFP-modified hFTN (hFTN-RFP) was synthesized through the genetic fusion of the N-terminus of RFP (model tumor antigen) to the C-terminal E-helix of hFTN subunit (human ferritin heavy chain), affording dense display of RFP on the surface of hFTN and efficient antigen exposure to immune cells in LNs (Fig. 4a,b). Although native hFTN with a diameter of 12 nm has “turn-inside” conformation where the C-terminal E-helix of hFTN subunit is located inside the cavity of particle, genetic linkage of recombinant protein or peptide to the C-terminus of hFTN subunit can make the engineered hFTN adopt “turn-inside-out” conformation where the genetically modified C-termini point outside of the particle upon self-assembly of 24 subunits (Fig. 4b)^29^,^34^. We also inserted a flexible glycine-rich linker peptide (G_3_SG_3_TG_3_SG_3_) between the E-helix of hFTN subunit and the N-terminus of RFP, which might provide further conformational flexibility to induce turn-inside-out conformation of the engineered hFTN (Fig. 4a). The C-terminal poly-histidine tag (His_6_) was added for Ni^2+^-affinity purification of hFTN-RFP from crude cell lysates. The successful synthesis of hFTN-RFP was confirmed through SDS-PAGE, DLS, and TEM analysis of the purified hFTN-RFP, showing the formation of homogeneous particles with a diameter of 22.0 ± 0.7 nm (Fig. 4b, Supplementary Fig. 5). Compared to the size of hFTN (Table 1), the increased diameter of hFTN-RFP is due to the additional surface-display of recombinant polypeptides, i.e. linker-RFP-His_6_. Importantly, Fig. 4c shows that after injected into the forepaw pad of healthy mice (100 μl, Supplementary Fig. 6), the Cy5.5-labeled hFTN-RFP showed a similar pattern of LN targeting through lymphatic vessels (white arrows) and accumulation (white dotted circles), as compared to the injection of RFP-free hFTN (Fig. 3c). The subcutaneously injected hFTN-RFP rapidly migrated from injection site to LN within 1 min, and the LN NIRF signals lasted up to 72 h, indicating the excellent LN targeting and prolonged retention of hFTN-RFP in LN, compared to the RFP-only injection (Fig. 4d).

### Vaccination of hFTN-RFP and immunotherapy of RFP-expressing tumor in mice

**Figure 5:**
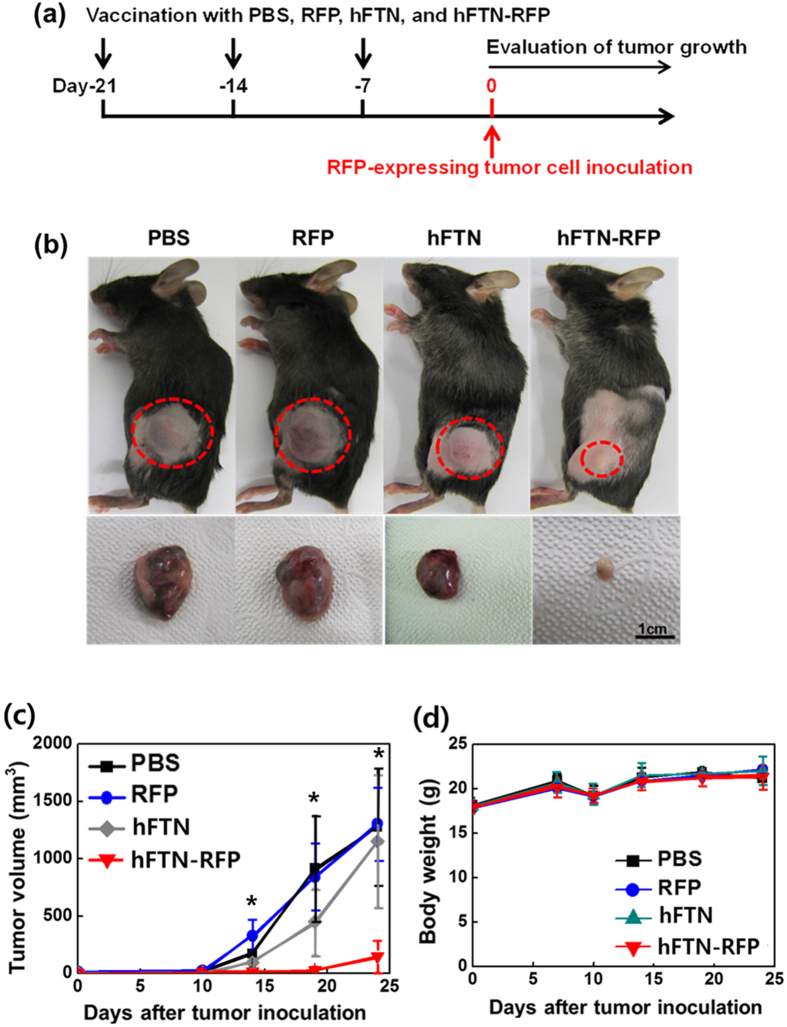
*In vivo* tumor inhibition effect of hFTN-RFP. (**a**) Vaccination schedule. [C57BL/6 mice were subcutaneously vaccinated with 10 μl of PBS (control), RFP (10 μM), hFTN (10 μM), and hFTN-RFP (10 μM) three times with 1-week interval. 1 week after the final vaccination, the mice were subcutaneously inoculated with RFP-expressing B16F10 melanoma cells (5 × 10^5^) into the flank.] (**b**) RFP-expressing tumor-bearing mice vaccinated with PBS, RFP, hFTN, or hFTN-RFP (top) and tumors excised from each mice (bottom). (Dotted circles in the top photos indicate tumors). (**c**) Time-course change of tumor volume of mice (**b**) after inoculation with RFP gene-transfected murine melanoma cells. (Asterisks in **c** indicate *p* < 0.05 between hFTN-RFP group and the other groups). (**d**) The body weight change of mice (**b**) (n = 5) after the inoculation of RFP-expressing B16F10 melanoma cells.

To estimate the effect of hFTN-RFP vaccination on tumor growth inhibition *in vivo*, C57BL/6 mice were subcutaneously injected with hFTN-RFP (10 μM), RFP (10 μM), hFTN (10 μM), or PBS, three times with 1-week interval, followed by the inoculation of RFP-expressing B16F10 melanoma tumor (Fig. 5a). At 19^th^ day after the tumor inoculation, the size of excised tumor treated with hFTN-RFP was obviously much smaller than the tumor in the RFP-, hFTN-, and PBS-treated mice (Fig. 5b). Time-course measurement of tumor volume shows that the RFP-, hFTN-, and PBS-treated tumor continuously grew to the large size exceeding 1000 mm^3^ (*p* < 0.05) for 24 days post injection (Fig. 5c). Vaccination with hFTN-RFP significantly enhanced the survival of tumor-bearing mice (Supplementary Fig. S7). All the mice treated with PBS or RFP died at 32^nd^ day after the tumor inoculation, and the mice vaccinated with hFTN-RFP exhibited a 60% survival rate when survival was monitored up to 36 days after the tumor inoculation. For all the mice tested, the difference in body weight was negligible during the entire period of tumor growth estimation, suggesting no toxic effect by RFP, hFTN, PBS, and hFTN-RFP injected to mice (Fig. 5d).

**Figure 6:**
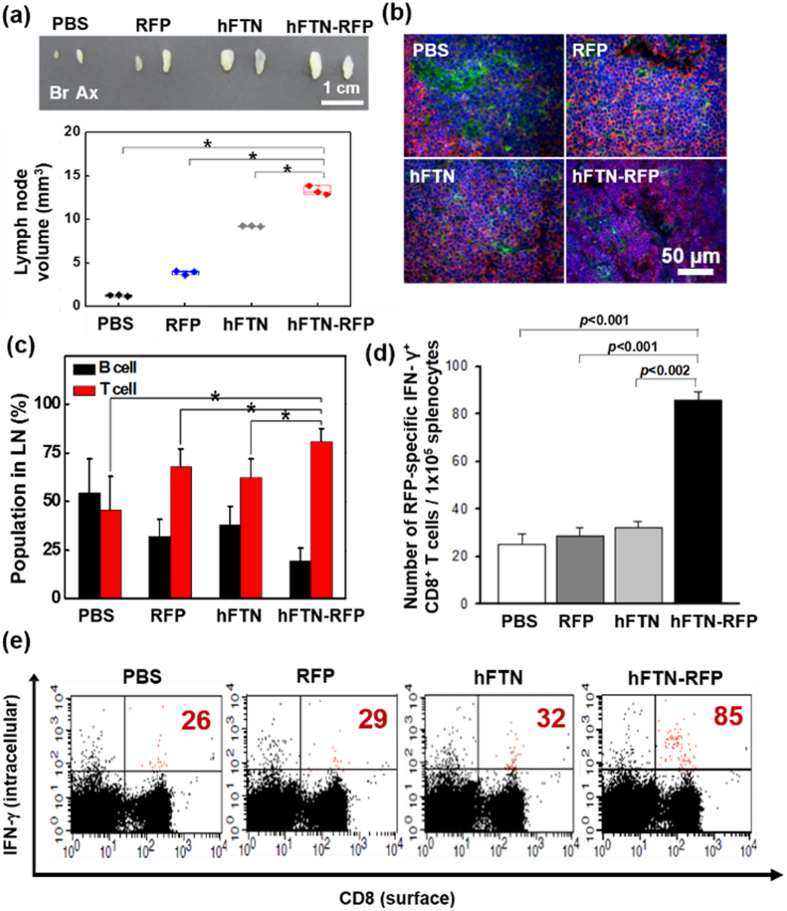
*In vivo* immune responses to vaccination of hFTN-RFP. (**a**) Photographic images of proximal LNs excised from the mice at 7^th^ day after final vaccination (top) and the volume of excised LNs (*n* = 3) (bottom). (Asterisks indicate *p* < 0.05 between hFTN-RFP group and the other groups). (**b**) Immunofluorescence double-staining of LNs in the mice vaccinated with PBS, RFP, hFTN, and hFTN-RFP to identify the lymphocyte population in the LNs. [The LNs are dual-stained with monoclonal mouse anti-human CD79α (green) for B cell detection and polyclonal rabbit anti-human CD3 (red) for T cell detection. The nucleus was stained with DAPI (blue).] (**c**) Relative population of the lymphocyte cells in the LNs of (**b**) from various mice (n = 6). (Asterisks indicate *p* < 0.05 between T cell population of hFTN-RFP group and T cell populations of the other groups). (**d**) The numbers of the CD8^+^ IFN-γ^+^ cell fractions from various groups of (**e**). (**e**) The strength of the CD8^+^ T cell response assessed by analyzing the frequency of intracellular IFN-γ-secreting CD8^+^ T cells in the spleen. [At 7^th^ day after final vaccination, splenocytes of mice injected with PBS, RFP, hFTN, and hFTN-RFP were activated *ex vivo* with RFP-derived peptide (S111 to I119/SSLQDGCFI). Double positive cells (CD8^+^ IFN-γ^+^) were then identified, and the numbers of the CD8^+^ IFN-γ^+^ cells was shown (red).].

The immune response of mice was also estimated by measuring the changes in LN volume in the mice that were vaccinated with PBS, RFP (10 μM), hFTN (10 μM), and hFTN-RFP (10 μM), three times with 1-week interval (Fig. 6a). For 1 week after the final vaccination, the average volume of LNs increased to 3.89 mm^3^, 9.23 mm^3^, and 13.31 mm^3^ in the mice treated with RFP, hFTN, and hFTN-RFP, respectively, as compared to PBS-treated LNs (1.26 mm^3^) (*p* < 0.05), indicating that the largest increase of LN size was caused by the hFTN-RFP vaccination. Furthermore, the lymphocyte population in LNs was comparatively analyzed using immunofluorescence double-staining of the LNs vaccinated with PBS, RFP, hFTN, and hFTN-RFP (Fig. 6b). The cell population in the regional LNs of PBS-treated mice comprised 54.4 ± 17.4% of B cells and 45.6 ± 17.4% of T cells, while the T cell population in LNs of the mice vaccinated with hFTN, RFP, and hFTN-RFP was 62.1 ± 9.7%, 68 ± 8.9%, and 78.9 ± 7.9%, respectively (*p* < 0.05), indicating that the hFTN-RFP vaccination increased the T cell populations in LNs to the highest level (Fig. 6c). (In case of the vaccination of hFTN-RFP, the actual number of B cells almost unchanged, while the number of T cells dramatically increased, which results in the noticeable change in the percentage of B and T cell population). To investigate whether hFTN-RFP can elicit the RFP-specific anti-tumor immune responses, the strength of CD8^+^ T cell response was assessed in the C57BL/6 mice that were subcutaneously vaccinated with hFTN-RFP (10 μM), RFP (10 μM), hFTN (10 μM), and PBS, three times with 1-week interval. At 7^th^ day after the final vaccination, splenocytes were activated *ex vivo* with RFP-derived peptide (S111 to I119 or SSLQDGCFI), which acts as an epitope for RFP-primed response^35^. The strength of the CD8^+^ T cell response was assessed by analyzing the frequency of intracellular interferon (IFN)-γ-secreting CD8^+^ T cells in the spleen (Fig. 6d). The number of IFN-γ-secreting CD8^+^ T cells in the spleen of the mice vaccinated with hFTN-RFP (85 ± 4) was approximately 3-fold higher, compared to the mice vaccinated with PBS (26 ± 5), RFP (29 ± 4), and hFTN (32 ± 3) (Fig. 6e; *p* < 0.002), indicating that hFTN-RFP stimulates most effectively the production of RFP-specific cytotoxic T cells. This suggests that tumor-specific antigen (RFP)-primed and anti-tumor immune response was provoked by hFTN-RFP.

## Discussion

The efficacy of four different protein nanoparticles in targeting LNs was carefully evaluated after they were labeled by NIRF dye (Cy5.5) and subcutaneously injected into mice. The Cy5.5 labeling of protein nanoparticles was done through simple chemical conjugation to surface-exposed amines on protein nanoparticles for imaging-based monitoring of LN targeting performance. From the LN imaging data, it is worth noting that hFTN rapidly migrated to the LNs with the short incubation time (less than 1 min), and the accumulation of hFTN in the LNs lasted for a sufficiently long period of time (6 day), compared to other protein nanoparticles. This rapid localization and prolonged accumulation of hFTN is presumably due to interaction between human ferritin and T lymphocytes, because human ferritin strongly binds to T cell immunoglobulin and mucin domain-2 (TIM-2), one of TIM family with important regulatory function of cellular immunity, which is expressed primarily on T cells and on subsets of B lymphocytes that are abundant in LNs^36^,^37^,^38^. Reportedly, binding of human ferritin to T cells induces the internalization of human ferritin into cellular endosomes, followed by eventually entering a lysosomal compartment^39^,^40^, which is distinguished from classical transferrin pathway for cellular iron delivery. The prolonged LN retention of hFTN is highly advantageous as an antigen carrier because antigens can be sufficiently exposed to immune cells in LNs. Recent studies suggest that several-day exposure of antigen and adjuvant to immune system amplifies the immunogenicity of a vaccine^10^,^41^,^42^.

In general, protein nanoparticles are spontaneously disassembled in living organisms and then degraded or removed through renal excretion, showing excellent biocompatibility. Moreover, hFTN is a human protein nanoparticle and therefore seems to have significant advantages over synthetic nanoparticles in terms of *in vivo* biocompatibility. The rapid migration of hFTN to and its prolonged accumulation in LNs may enable hFTN to be used as a contrast agent to delineate sentinel LNs for resection and appropriate biopsies as well as TSA-carrying vaccine platform. Sentinel LNs are the first LNs that cancer cells reach after begin to migrate from primary tumor through lymphatic vessels^43^. Following identification of sentinel LNs using contrast agents, the sentinel LNs are surgically removed and immediately examined using standard pathologic techniques to diagnose cancer metastasis^44^,^45^. Thus, rapid, sensitive, and accurate identification of sentinel LNs would be of crucial importance in determining cancer treatment protocols to eradicate tumor or prevent further metastasis. The labeling of hFTN using an NIRF dye with fluorescence spectrum of 650–900 nm, which has relatively low auto-fluorescence and capability of high tissue penetration, would allow imaging-based sensitive detection of even deep-tissue LNs^46^,^47^.

The well-oriented and flexible surface architecture of hFTN was effectively engineered to expose tumor antigens on its surface through genetic modification of C-terminus of hFTN subunit. That is, due to the conformational flexibility of C-terminal E-helices of native hFTN, native turn-inside conformation of hFTN was converted to recombinant turn-inside-out conformation by genetic fusion of model TSA (RFP) to the C-terminus of hFTN subunit, where 24 RFPs per hFTN particle are located outside the particle. The genetic modification of hFTN subunits with other tumor antigens should not hamper their self-assembly activity, which is subject to depend on size and conformation of tumor antigens to be genetically attached to the hFTN subunit. As described in this work, a flexible glycine-rich linker peptide (G_3_SG_3_TG_3_SG_3_ in this study) that is placed between the C-terminus of hFTN subunit and tumor antigen may allow further the conformational flexibility to retain the self-assembly activity of the engineered hFTN subunits. This suggests that hFTN can be used as a 3D scaffold to carry and directly deliver tumor-specific proteins or peptides to LNs and therefore mediate efficient cancer immunotherapy. As compared with the vaccination of RFP only, the genetically engineered hFTN (i.e. hFTN-RFP) that densely presents RFPs on the hFTN surface effectively targeted LNs and stayed in the LNs for a sufficiently long period of time, significantly increased the T cell population in LNs, induced strong and antigen-specific CD8^+^ T cell response, and successfully inhibited the RFP-expressing tumor growth based on its antigen-specific anti-tumor activity. Although RFP was used as a model tumor antigen for proof-of-concept in this study, it can be switched to other tumor antigens. In future, actual tumor antigen-containing hFTN also needs to be evaluated through treating existed tumors to show that the effectiveness of our cancer vaccines is whether in eliminating existed tumor or in protecting tumor emergence and growth. This approach to cancer immunotherapy using genetically engineered hFTN with enhanced tumor-specific immunogenicity seems to have a great potential in clinically successful cancer treatment.

## Methods

### Biosynthesis and characterization of protein nanoparticles (DPS, PTS, HBVC, hFTN, and hFTN-RFP) used for LN targeting and cancer immunotherapy

Through polymerase chain reaction (PCR) amplification using the appropriate primers, the six gene clones were prepared using a previously cloned expression vector^24^,^26^,^30^, encoding NH_2_-*Nde*I-(His)_6_-DPS-*Hin*dIII-COOH, NH_2_-*Nde*I-PTSα-*Hin*dIII-COOH, NH_2_-*Nde*I-PTSβ-(His)_6_-*Hin*dIII-COOH, NH_2_-*Nde*I-HBVC-*Hin*dIII-COOH, NH_2_-*Nde*I-(His)_6_-hFTN-*Hin*dIII-COOH, and NH_2_-*Nde*I-(His)_6_-hFTN-*Xho*I-linker (G_3_SG_3_TG_3_SG_3_)-RFP-*Hin*dIII-COOH, and sequentially ligated into pT7-7 or pET-28a(+). plasmid to construct the following expression vectors: pT7-DPS, pT7-PTSβ, pET-28a(+)-PTSα, pET-28a(+)-HBVC, pT7-FTN, and pT7-FTNR, respectively (Supplementary Fig. 1). After complete sequencing, *E. coli* BL21 (DE3) was transformed with each of pT7-DPS, pET-28a(+)-HBVC, pT7-FTN, and pT7-FTNR, and ampicillin- or kanamycin-resistant transformants were selected and used to synthesize the protein nanoparticles, DPS, HBVC, hFTN, and hFTN-RFP, respectively. To synthesize the protein nanoparticle PTS, *E. coli* BL21 (DE3) was co-transformed by both of two recombinant expression vectors, pET-PTSα and pT7-PTSβ, and both kanamycin- and ampicillin-resistant transformants were finally selected. The detailed procedures for recombinant gene expression and purification of synthesized protein nanoparticles are well described in our previous reports^30^,^48^,^49^,^50^,^51^.

Size distribution and surface charge of the prepared protein nanoparticles were measured in triplicate using a Zetasizer Nano ZS (Malvern Instruments,Ltd., Worcestershire, UK) equipped with a 633 nm wavelength of laser. Each sample was dissolved in distilled water at concentration of 1.0 mg/ml and sonicated for 3 min by probe-equipped sonicator (Ultrasonic Processor, GEX-600; Sonics & Materials, Newtown, CT) at 90 W. The morphological shapes of protein nanoparticles were analyzed using TEM (CM-200 electron microscope, Philips, CA), operating at an acceleration voltage at 80 kV. For the preparation of TEM samples, one drop of each protein nanoparticle suspension (1 mg/ml) was placed onto a 200-mesh copper grid which was pre-coated with carbon. After 2 min of deposition, distilled water was removed by air drying. Negative staining was applied using a droplet of a 2% (w/v) aqueous uranyl acetate solution.

### NIR fluorescence labeling of protein nanoparticles

For *in vivo* imaging of LNs, NIR dye, Cy5.5 was used to label the five different protein nanoparticles (DPS, PTS, HBVC, hFTN, and hFTN-RFP) and RFP. 2 mmol of Cy5.5 N-hydroxysuccinimide (NHS) ester (Cy5.5-NHS, GE Healthcare, Piscataway, NJ) with excitation and emission maximum wavelength of 675 and 693 nm, respectively, was incubated with the purified protein nanoparticles in sodium bicarbonate (0.1 M, pH 8.5) at room temperature for 12 h. Cy5.5-labeled protein nanoparticles were loaded onto a sucrose step gradient (40, 35, 30, 25, and 20%w/v) and centrifuged at 35,000 rpm for 16 h at 4 °C to separate the unbound Cy5.5 from Cy5.5-labeled protein nanoparticles or RFP. Subsequently, sucrose solution (20–25% sucrose) containing the Cy5.5-labeled protein nanoparticles or RFP was fractionated and then exchanged to PBS (2.7 mM KCl, 137 mM NaCl, 2 mM KH_2_PO_4_, 10 mM Na_2_HPO_4_, pH 7.4) by ultrafiltration (Amicon Ultra 100K, Millipore, Billerica, MA).

### *In vivo* and *ex vivo* NIR image analyses

All experiments using live animals were carried out in compliance with the relevant laws and institutional guidelines of Korea University and Korea Institute of Science and Technology (KIST). The Korea Institute of Science and Technology Animal Ethics Committee approved the use of animals in this study (2013-01-025), and all animal experimental procedures were in compliance with the institutional guidelines of Korea Institute of Science and Technology and the relevant laws. All protocols that synthesize protein nanoparticles for all experiments were synthesized in Korea University. Male athymic nude mice of age 5 weeks (Orient, Korea) were anesthetized by injecting xylazine (10 mg/kg body weight) and Zolazepam (5 mg/kg body weight) intraperitoneally. DPS, PTS, HBVC, hFTN, RFP, and hFTN-RFP in PBS (100 μl) were subcutaneously injected into the right footpad of mice (Supplementary Fig. 2 and 6). *In vivo* NIR images were acquired with a Kodak image station (4000 MM; Kodak, New Haven, CT) equipped with a Cy5.5 bandpass emission filter and a special C-mount lens or IVIS spectrum imaging system (Caliper Life Sciences, Hopkinton, MA). For the quantitative analysis, total photon counts in the tumors were measured using the region of interest (ROI) tool. The mice were sacrificed, and their proximal and distal LNs were removed and subjected to NIR fluorescence imaging. To confirm the movement of protein nanoparticles into LNs through lymphatic vessels, real-time intravascular dynamics of Cy5.5-labeled hFTN or hFTN-RFP was observed in live mice using a Small Animal Imaging System with 620–650 nm/680–710 nm (excitation/emission) channel. Prior to recording, the mice skin around the LN was removed to develop clear flow image, and 100 μl of Cy5.5-labeled hFTN or hFTN-RFP was subcutaneously injected at the right footpad of mice. After the *in vivo* NIR fluorescence imaging, the mouse was sacrificed, and liver, lung, spleen, kidney, heart, and/or LNs were excised and imaged *ex viv*o with a Kodak image station. For the NIR fluorescence imaging of Cy5.5-labeled hFTN delivered to LNs, excised LNs were embedded in to an optimal cutting-temperature (OCT) compound for cryosection preparation. Frozen sections (8 μm-thick) cut from the OCT-embedded specimens were stained with 4,6-diamidino-2-phenylindole (DAPI) and observed with an IL-70 microscope (Olympus) equipped with a mercury arc lamp, a digital camera, and optical filter sets for fluorescence imaging of DAPI and Cy5.5 fluorescence.

### Histological and immunofluorescence analysis of LNs

After the three-time vaccination of PBS, RFP, hFTN, or hFTN-RFP, the LN tissues were fixed in neutral buffered formalin and embedded in paraffin for immunofluorescence double-staining. The tissue slides (5 μm-thick) were deparaffinized and rehydrated, and the antigens were retrieved in boiling Tris-EDTA buffer (pH 9.0) for 10 min. The slides were first incubated in monoclonal mouse anti-human CD79α (1:200 diluted in PBS; DakoCytomation, Carpinteria, CA) for 2 h, and the fluorescein isothiocyanate (FITC)-labeled goat anti-mouse IgG2b (1:300 diluted in PBS; Santa Cruz biotechnology, Santa Cruz, CA) was treated for 40 min for B cell detection. Then, polyclonal rabbit anti-human CD3 (1:200 diluted in PBS; DakoCytomation) and CFL555-labeled mouse anti-rabbit IgG (1:300 diluted in PBS; Santa Cruz biotechnology) was applied in consecutive order for T cell detection. The slides were covered with permount mounting medium containing DAPI for nuclear stain, and the fluorescence images were acquired using IX81-ZDC focus drift compensation microscope and digital image transfer software (Olympus). Eight random fields (×200 magnification) were selected in each slide, and the B and T cells were counted using imaging analysis software (Image Pro Plus 4.1; Media Cybernetics, Silver Spring, MD).

### Cytokine-secreting CD8^+^ T cell assay

C57BL/6 mice of age 6 weeks (Orient, Korea) were subcutaneously vaccinated with 10 μl of hFTN-RFP (10 μM), hFTN (10 μM), RFP (10 μM), PBS three times with 1-week interval. One week after the final vaccination, spleen was excised, and splenocytes were harvested from the spleen using cell strainer. Splenocytes were treated with red blood cell lysis buffer, incubated for 1 min at room temperature, and washed with PBS. Splenocytes (5 × 10^6^ cells/ml) from each vaccinated mouse were activated for 1 h with 1 μg/ml of RFP-derived peptide (S111 to I119 or SSLQDGCFI)^35^ that acts as an epitope for RFP-primed response. Cells were stained with CD8 antibody (BD Biosciences, San Jose, CA). Then, intracellular IFN-γ staining was performed with the Cytofix/Cytoperm (BD Biosciences) using IFN-γ antibody (BD Biosciences). IFN-γ-secreting CD8^+^ T cells were characterized by fluorescence-activated cell sorting (FACS) analysis and were quantified using CELLQuest software.

### Estimation of tumor growth inhibition

For estimating the effect of tumor growth inhibition, C57BL/6 mice of age 6 weeks (Orient, Korea) were subcutaneously vaccinated with 10 μl of hFTN-RFP (10 μM), hFTN (10 μM), RFP (10 μM), and PBS three times with 1-week interval. One week after the final vaccination, the mice were subcutaneously inoculated with RFP gene-transfected mouse melanoma (B16F10) cells into the flank. RFP-expressing B16F10 cells were established via liposome-mediated RFP gene transfection method^52^. In brief, 5 x 10^4^ of B16F10 cells were seeded into 6-well plates with RPMI 1640 media, incubated in humidified 5 % CO_2_ incubator for 24 h at 37 ^o^C, and washed using Opti-MEM (Gibco, Grand Island, NY) before the RFP transfection. Then Lipofectamine/pDSRed2 complex that was prepared by gently mixing pDSRed2 (1 μg) and lipofectamine (1 μg, Invitrogen, Carlsbad, CA) in 100 μl of Opti-MEM was stabilized for 20 min at 37 °C and then B16F10 cells were incubated with the lipofectamine/pDSRed2 complex in humidified 5% CO_2_ incubator for 4 h at 37 °C. Culture media were then changed to RPMI 1640 media to allow cells to recover for 48 h. To isolate transfected cells form non-transfected cells, selection media containing G418 (1 mg/ml, Gibco, Grand Island, NY) was added into the RPMI 1640 media for 2 days, and this media was changed weekly. Selected RFP-expressing B16F10 (RFP-B16F10) cells were cultured in RPMI 1640 containing 25 mM HEPES and G418 (0.5 mg/ml) at 37 °C in humidified 5% CO_2_, and after 48-h cultivation, the 5 × 10^5^ RFP-B16F10 cells were inoculated into the mice. Tumor size was observed by measuring the minor and major axes of the tumors with electronic digital callipers. Tumor volume was calculated according to the following formula^53^: (tumor volume) = (major axis) × (minor axis)^2^ × 0.52. All mice were euthanized after tumor volume of control group reached 1500 mm^3^. Body weight of mice was also monitored.

### Statistical analysis

Data represents the means ± standard deviation. A one-way analysis of variance was used for the comparison of variables between groups.

## Additional Information

**How to cite this article**: Lee, B.-R. *et al.* Engineered Human Ferritin Nanoparticles for Direct Delivery of Tumor Antigens to Lymph Node and Cancer Immunotherapy. *Sci. Rep.* **6**, 35182; doi: 10.1038/srep35182 (2016).

## Supplementary Material

Supplementary Information

## Figures and Tables

**Figure 1:**
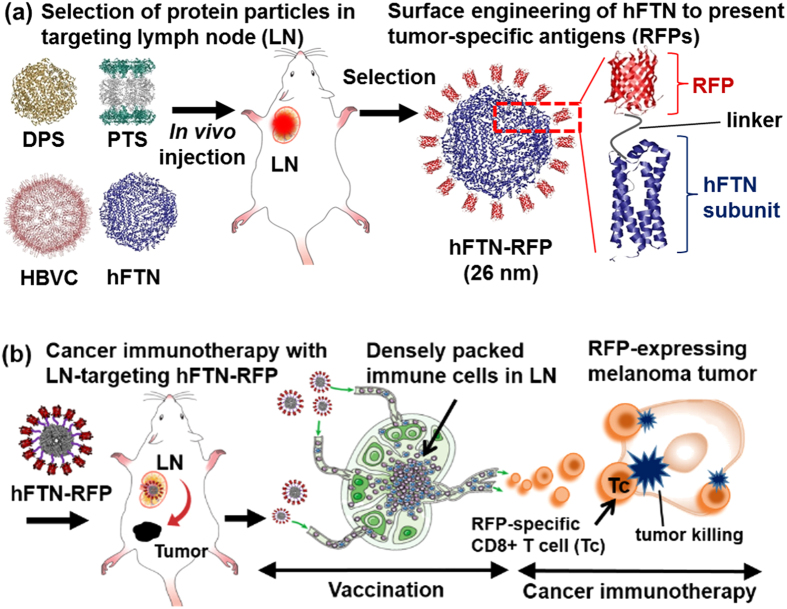
Schematic illustration of LN targeting-based cancer immunotherapy using protein nanoparticles as platform of tumor-specific antigen carrier. (**a**) Through the evaluation of LN-targeting ability of four candidate protein nanoparticles, human ferritin heavy chain (hFTN), *T. acidophilum* proteasome (PTS), hepatitis B virus capsid (HBVC), and *E. coli* DNA binding protein (DPS), hFTN was selected as LN-targeting carrier of tumor-specific antigen (RFP) for cancer immunotherapy. (**b**) Vaccination using hFTN-RFP through direct LN targeting, followed by immunotherapy of RFP-expressing tumor by RFP-specific CD8^+^ T cells.

**Table 1:** Molecular weight, diameter and zeta potential of 4 different protein NPs (DPS, PTS, HBVC, and hFTN).

Protein NP	Molecular weight (kD)^a^	Diameter (nm)^b^	Zeta potential (mV)^c^
DPS	237.12	9.5 ± 1.2	−5.63 ± 0.33
PTS	695.66	13.4 ± 2.1	−2.13 ± 0.27
HBVC	4147.20	32.3 ± 1.9	−7.50 ± 0.43
hFTN	525.84	11.74 ± 0.8	−5.69 ± 0.44

^a^Average molecular weight from EXPASY.

^b^Mean diameter measrued by dynamic light sattering (DLS).

^c^Zeta potential measured by DLS.
