# Supplementary material for: Engineered Human Ferritin Nanoparticles for Direct Delivery of Tumor Antigens to Lymph Node and Cancer Immunotherapy
Source: Sci Rep. 2016 Oct 11;6:35182. doi: 10.1038/srep35182 (PMC5057094; doi:10.1038/srep35182)
Supplement: Supplementary Information [file srep35182-s1.doc]

Supporting Information

Engineered Human Ferritin Nanoparticles for Direct Delivery of Tumor Antigens to Lymph Node and Cancer Immunotherapy

Bo-Ram Lee**^¶^**, Ho Kyung Ko**^¶^**, Ju Hee Ryu, Keum Young Ahn^†^, Young-Ho Lee, Se Jin Oh,

Jin Hee Na, Tae Woo Kim, Youngro Byun, Ick Chan Kwon,

Kwangmeyung Kim*****, and Jeewon Lee*****

¶ These authors contributed equally to this work

[*]Prof. J. Lee(Corresponding author), B.-R. Lee, K. Y. Ahn

Department of Chemical and Biological Engineering, College of Engineering,

Korea University, Anam-Ro 145, Seoul 136-713 (Republic of Korea)

E-mail:leejw@korea.ac.kr

[*]Dr. K. Kim(Co-corresponding author), H. K. Ko, J. H. Ryu, J. H. Na, I. C. Kwon

Center for Theragnosis, Biomedical Research Institute, Korea Institute of Science and Technology, 39-1 Hawolgok-dong, Seongbuk-gu, Seoul 136-791 (Republic of Korea)

E-mail:kim@kist.re.kr

Prof. T. W. Kim, Y.-H. Lee, S. J. Oh

Division of Infection and Immunology, Graduate School of Medicine,

Korea University, Anam-Ro 145, Seoul 136-713 (Republic of Korea)

Y. Byun, H. K. Ko

Department of Molecular Medicine and Biopharmaceutical Sciences,

Graduate School of Convergence Science and Technology, Seoul National University, Seoul 151-742 (Republic of Korea)

^†^ Present address: R&D Division, Celltrion Inc., Songdo-dong 13-1, Yeonsu-gu, Incheon, 406-840, Republic of Korea.

**Supplementary Figures**

**Figure S1.**

**Figure S1.** Result of SDS-PAGE analysis of the purified proteinticles, DPS, PTS, HBVC, and hFTN (M, pre-stained protein ladder).

**Figure S2.**

**
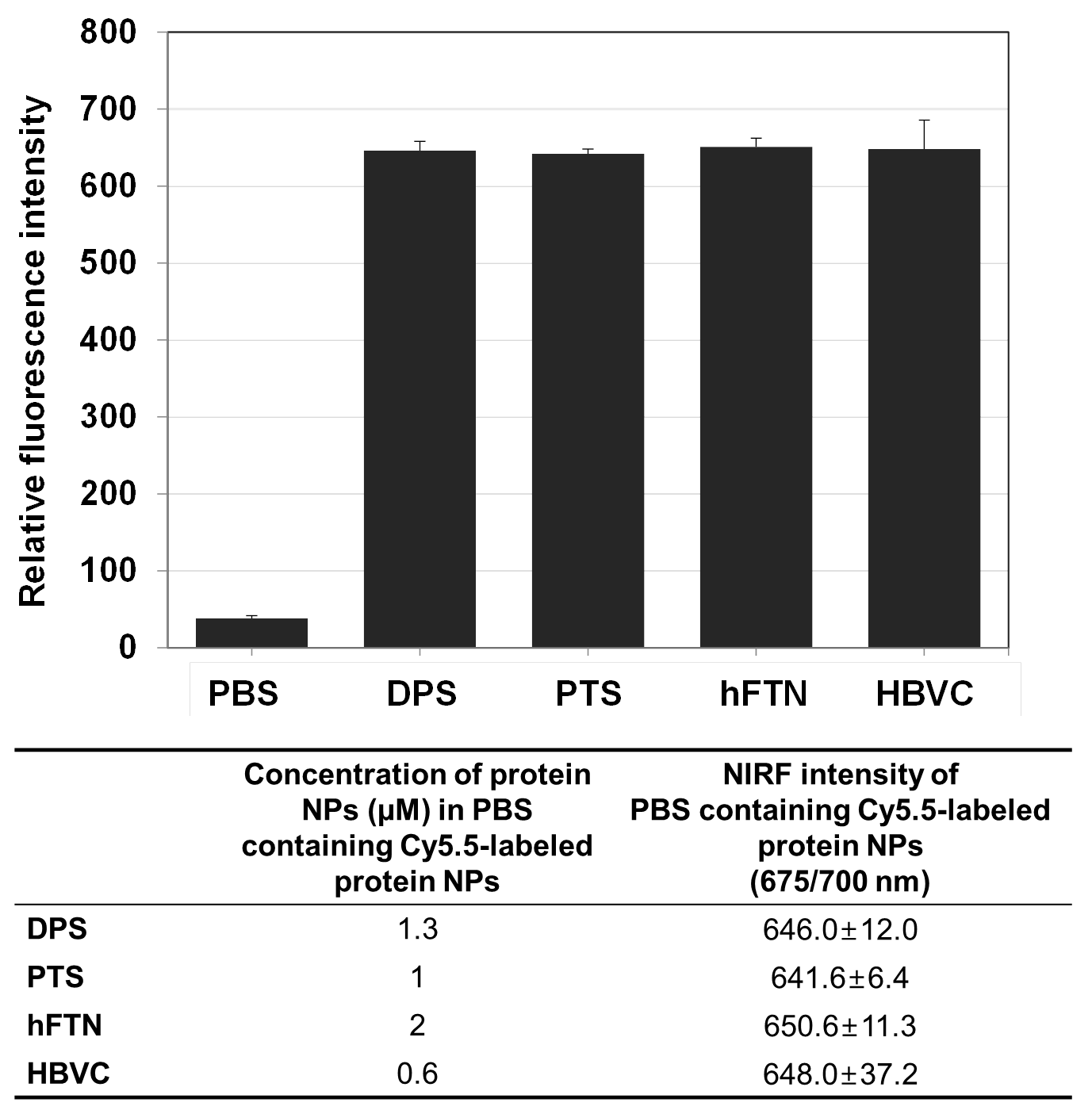
**

**Figure S2.** Concentration of protein NPs (DPS, PTS, hFTN, and HBVC) in and NIRF intensity of PBS solutions that are prepared to estimate LN-targeting efficiency of protein NPs, containing Cy5.5-labeled protein NPs.

**Figure S3.**

**(a)**

**(b)**

**Figure S3. (a)** *In vivo* near-infrared (NIR) fluorescence images acquired with an IVIS spectrum imaging system at pre-determined time points for 1 h. [Through the footpad of C57BL/6 mice, each of PBS and four candidate proteinticles (DPS, PTS, HBVC, and hFTN) that were labeled by Cy5.5 was subcutaneously injected.] **(b)** Time-course NIR fluorescence intensity of the LN region of C57BL/6 mice of **a**.

**Figure S4.**

**(a)**

**
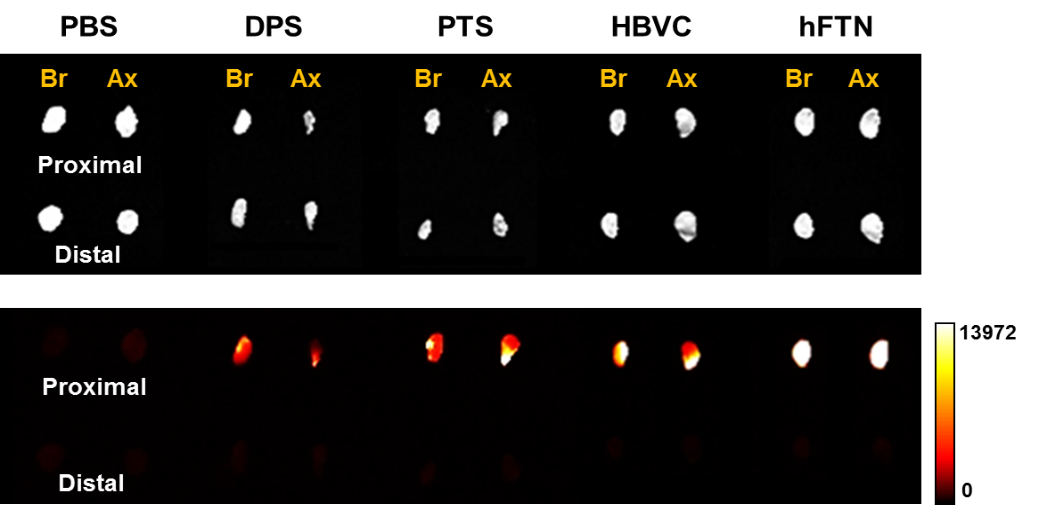
**

**(b)**

**
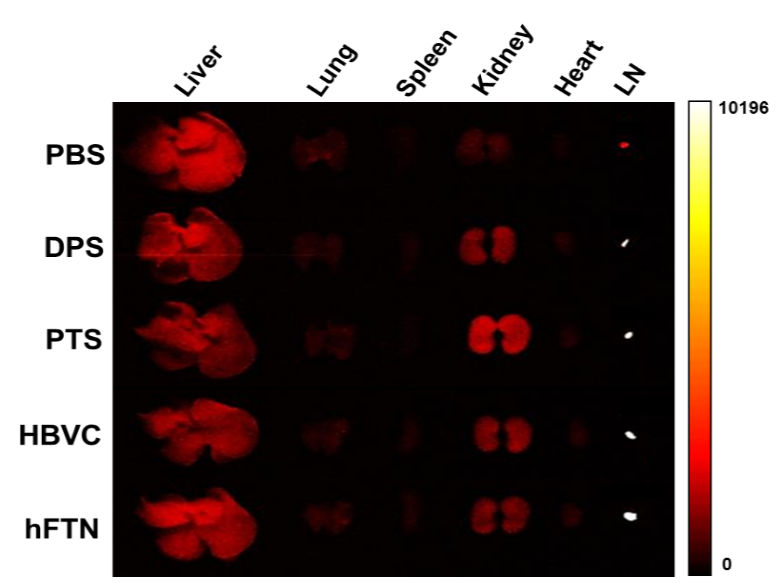

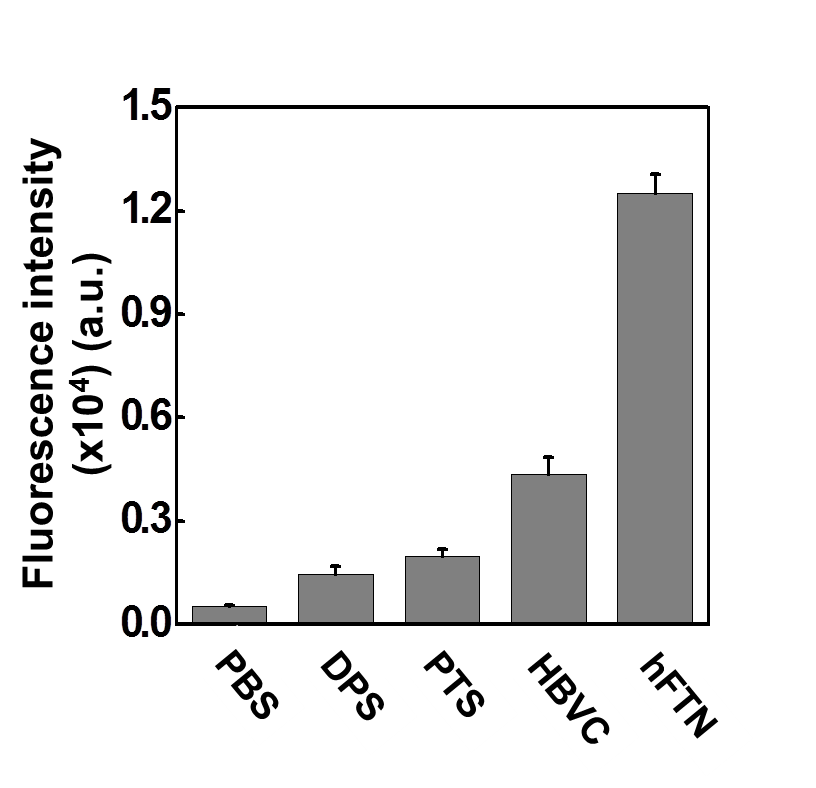
**

**(c)**

**(d)**

**
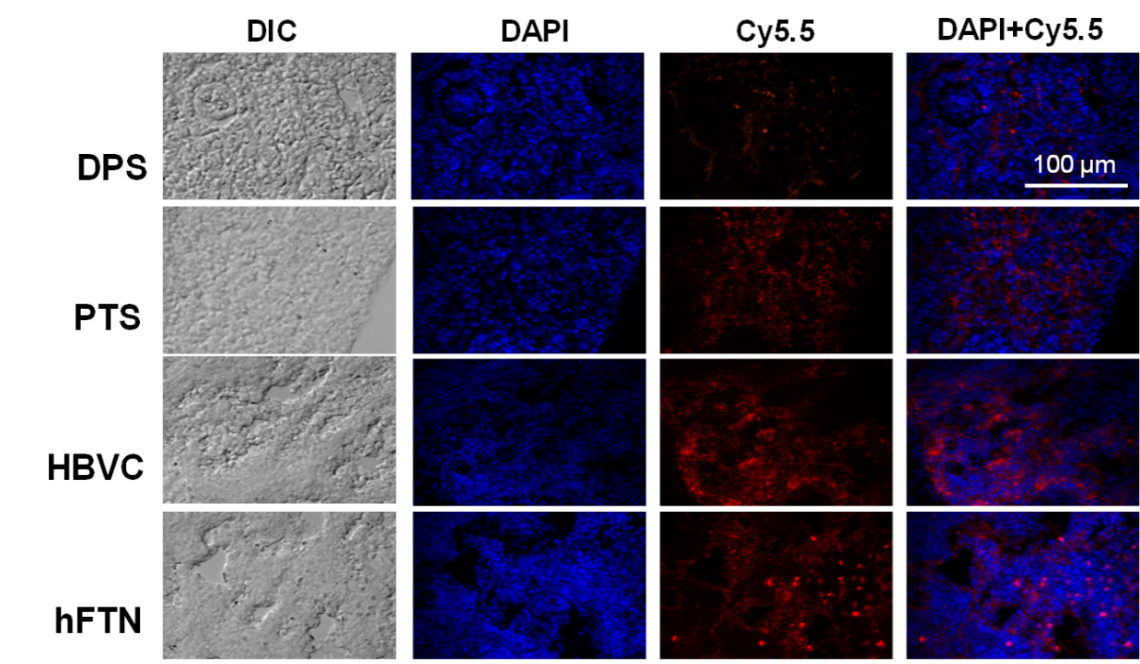
**

**Figure S4. (a)** Photo images (top) and NIR fluorescence images (bottom) of excised proximal (injection side) and distal LNs [i.e. respective brachial (Br) and axillary (Ax) LNs] removed from the mice injected subcutaneously with PBS and four candidate proteinticles (DPS, PTS, HBVC, and hFTN) labeled with Cy5.5. **(b)** Fluorescence intensity in the proximal LN region of **a**. **(c)** *Ex vivo* fluorescence images of liver, lung, spleen, kidney, heart, LN of mice of **a**. **(d)** Fluorescence signals from the LN sections of mice of **a** after subcutaneous injection of four candidate proteinticles (red). The nucleus was stained with DAPI (blue). (The photo and NIR fluorescence images of **a** and **c** were acquired with a Kodak image station.)

**Figure S5.**

**(a)**


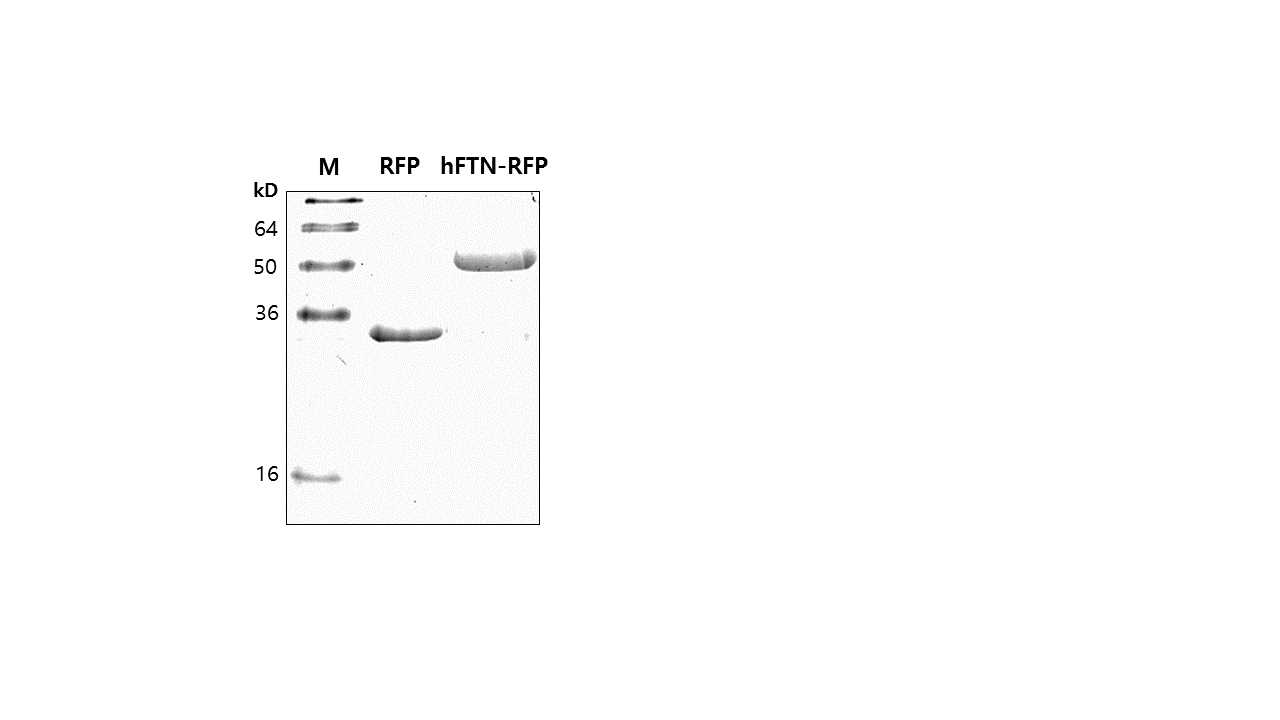


**(b)**

**Figure S5.** Result of **(a)** SDS-PAGE of purified RFP and hFTN-RFP tested in growth inhibition of RFP-expressing B16F10 melanoma tumor (M, pre-stained protein ladder) and **(b)** DLS analysis and TEM image of purified hFTN-RFP.

**Figure S6.**

**Figure S6.** Concentration of RFP or hFTN-RFP in and NIRF intensity of PBS solution that is prepared to estimate LN-targeting efficiency of RFP or hFTN-RFP, containing Cy5.5-labeled RFP or Cy5.5-labeled hFTN-RFP.

**Figure S7.**

**Figure S7.** The survival rate of mice. RFP-expressing tumor-bearing mice were vaccinated with PBS, RFP, hFTN, or hFTN-RFP with a vaccination schedule of Fig.5**a.**
